# Supplementary material for: Flavor Quality Analysis of Ten Actinidia arguta Fruits Based on High-Performance Liquid Chromatography and Headspace Gas Chromatography–Ion Mobility Spectrometry
Source: Molecules. 2023 Nov 13;28(22):7559. doi: 10.3390/molecules28227559 (PMC10674867; doi:10.3390/molecules28227559)
Supplement: Supplementary file 1 [file molecules-28-07559-s001.zip › molecules-2654078-supplementary.pdf]

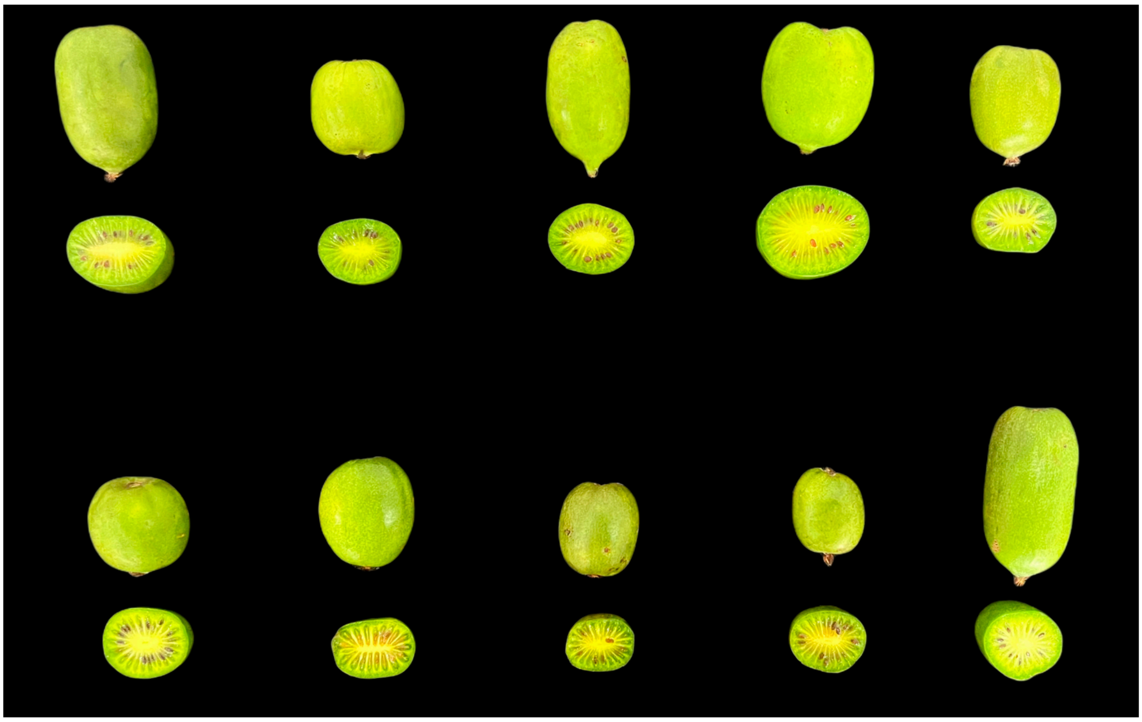

Figure S1. The pictures of ten *A. arguta* varieties used in this study. From left to right, the first column is 'Kuilv', 'Fenglv', 'Jialv', 'Wanlv', and 'Xinlv'; the second column is 'Pinglv', 'Lvbao', 'Cuiyu', 'Tianxinbao', and 'Longcheng No.2'.

Table S1. GC-IMS integration parameters of volatile compounds detected in *A. arguta* fruits.

Count		Compounds	CAS#	Formula	MW	RI	Rt [sec]	Dt [a.u.]
1	Aldehydes	(E)-2-Heptenal D	C18829555	C7H12O	112.2	1332.4	707.573	1.66732
2		(E)-2-Heptenal M	C18829555	C7H12O	112.2	1331.8	706.641	1.25852
3		(E)-2-Hexenal D	C6728263	C6H10O	98.1	1228.8	538.695	1.52704
4		(E)-2-Hexenal M	C6728263	C6H10O	98.1	1228	537.43	1.18512
5		(E)-2-Octenal D	C2548870	C8H14O	126.2	1428.1	857.246	1.81976
6		(E)-2-Octenal M	C2548870	C8H14O	126.2	1429.9	860.273	1.35715
7		(E)-2-Pentenal D	C1576870	C5H8O	84.1	1152.3	429.912	1.36561
8		(E)-2-Pentenal M	C1576870	C5H8O	84.1	1155.2	433.58	1.11013
9		(Z)-4-Heptenal D	C6728310	C7H12O	112.2	1217.6	521.002	1.61223
10		(Z)-4-Heptenal M	C6728310	C7H12O	112.2	1210.2	509.714	1.1513
11		1-Hexanal D	C66251	C6H12O	100.2	1095.1	364.304	1.56742
12		1-Hexanal M	C66251	C6H12O	100.2	1095.1	364.304	1.26313
13		1-Nonanal	C124196	C9H18O	142.2	1400.5	811.114	1.47214
14		3-Methyl butanal	C590863	C5H10O	86.1	936.1	262.201	1.40852
15		Butanal D	C123728	C4H8O	72.1	855.4	228.299	1.28537
16		Butanal M	C123728	C4H8O	72.1	865.6	232.302	1.09113
17		Diethyl acetal	C105577	C6H14O2	118.2	861.6	230.741	0.96562
18		Heptanal D	C111717	C7H14O	114.2	1197.9	491.341	1.69632
19		Heptanal M	C111717	C7H14O	114.2	1202.2	497.545	1.33061
20		Benzaldehyde	C100527	C7H6O	106.1	1531	1053.665	1.15214
21		Valeraldehyde	C123386	C3H6O	58.1	857.2	228.982	1.05585
22		Valeraldehyde D	C110623	C5H10O	86.1	1009.8	299.861	1.42345
23		Valeraldehyde M	C110623	C5H10O	86.1	1009	299.303	1.18328
24	Alcohols	(Z)-2-Penten-1-ol	C1576950	C5H10O	86.1	1341.2	720.076	0.94261
25		Isoamyl alcohol D	C123513	C5H12O	88.1	1215.2	517.283	1.49058
26		Isoamyl alcohol M	C123513	C5H12O	88.1	1215.8	518.215	1.24622
27		1-Hexanol D	C111273	C6H14O	102.2	1367.6	759.214	1.64083

28		1-Hexanol M	C111273	C6H14O	102.2	1366	756.824	1.32789
29		1-Hydroxy-2-propanone	C116096	C3H6O2	74.1	1265.4	600.856	1.04385
30		1-Octen-3-ol	C3391864	C8H16O	128.2	1475.8	943.203	1.16364
31		1-Pentanol	C71410	C5H12O	88.1	1262.4	595.534	1.257
32		1-Penten-3-ol	C616251	C5H10O	86.1	1167.3	449.188	0.94193
33		1-Propanol	C71238	C3H8O	60.1	1058	334.721	1.11065
34		3-Methyl-2-butanol	C598754	C5H12O	88.1	1054.6	332.154	1.44161
35		1-Butanol	C71363	C4H10O	74.1	1156.4	435.103	1.18347
36		2-Heptanol	C543497	C7H16O	116.2	1300.4	663.476	1.72087
37		Ethanol D	C64175	C2H6O	46.1	948.1	267.663	1.1318
38		Ethanol M	C64175	C2H6O	46.1	956	271.286	1.04416
39		Isobutanol D	C78831	C4H10O	74.1	1108.3	378.135	1.36557
40		Isobutanol M	C78831	C4H10O	74.1	1111.3	381.492	1.17065
41		1-Penten-3-one D	C1629589	C5H8O	84.1	1051	329.461	1.31154
42		1-Penten-3-one M	C1629589	C5H8O	84.1	1051.8	330.035	1.07599
43		Acetoin D	C513860	C4H8O2	88.1	1292.6	651.606	1.33997
44	Ketones	Acetoin M	C513860	C4H8O2	88.1	1293	652.538	1.05873
45		3-Octanone	C106683	C8H16O	128.2	1264	598.436	1.30462
46		Acetone	C67641	C3H6O	58.1	846.9	224.958	1.12006
47		Pentan-2-one	C107879	C5H10O	86.1	1009.6	299.71	1.36726
48	Acids	Acetic acid D	C64197	C2H4O2	60.1	1496.2	982.63	1.15499
49		Acetic acid M	C64197	C2H4O2	60.1	1499.8	989.798	1.0478
50		Ethyl acetate	C141786	C4H8O2	88.1	897.9	245.568	1.34375
51		Propyl acetate	C109604	C5H10O2	102.1	977.2	281.343	1.48148
52		Methyl benzoate	C93583	C8H8O2	136.1	1661.5	1368.998	1.20809
53		Ethyl butyrate D	C105544	C6H12O2	116.2	1044	324.193	1.55974
54		Ethyl butyrate M	C105544	C6H12O2	116.2	1055.2	332.588	1.21395
55		Butyl isovalerate D	C109193	C9H18O2	158.2	1296	657.749	1.89962
56		Butyl isovalerate M	C109193	C9H18O2	158.2	1295.2	656.625	1.39042
57		Butyl acetate D	C123864	C6H12O2	116.2	1087.8	358.258	1.61654
58		Butyl acetate M	C123864	C6H12O2	116.2	1055.4	332.788	1.23645
59		Ethyl formate D	C109944	C3H6O2	74.1	853.4	227.494	1.21473
60		Ethyl formate M	C109944	C3H6O2	74.1	849.8	226.113	1.07293
61		Geranyl formate	C105862	C11H18O2	182.3	1607.2	1227.62	1.22408
62	Esters	Hexyl acetate	C142927	C8H16O2	144.2	1241.5	559.45	1.38689
63		Hexyl propanoate	C2445763	C9H18O2	158.2	1302.4	666.173	1.42829
64		Isopentyl acetate D	C123922	C7H14O2	130.2	1154.6	432.847	1.74622
65		Isopentyl acetate M	C123922	C7H14O2	130.2	1153.5	431.446	1.30574
66		Isobutyl acetate	C110190	C6H12O2	116.2	1039.7	321.07	1.61897
67		Methyl isobutyrate	C547637	C5H10O2	102.1	838.7	221.818	1.1481
68		Methyl acetate	C79209	C3H6O2	74.1	854.2	227.809	1.19268
69		Methyl butanoate	C623427	C5H10O2	102.1	1039.4	320.805	1.14635
70		Pentyl acetate D	C628637	C7H14O2	130.2	1194.2	485.934	1.76536
71		Pentyl acetate M	C628637	C7H14O2	130.2	1195	487.079	1.3132
72		Ethyl propionate	C105373	C5H10O2	102.1	974.6	280.122	1.45551
73		Propyl propionate	C106365	C6H12O2	116.2	1056	333.196	1.587
74		α-Phellandrene	C99832	C10H16	136.2	1217.3	520.491	1.6603
75		α-Pinene	C80568	C10H16	136.2	1030.8	314.597	1.21616
76	Terpenes	β-Pinene	C127913	C10H16	136.2	1179.1	464.874	1.21947
77		Myrcene	C123353	C10H16	136.2	1126.7	398.949	1.21743
78		Terpinolene	C586629	C10H16	136.2	1293.8	653.954	1.21952
79	Pyrazines	2,3,5-Trimethylpyrazine	C14667551	C7H10N2	122.2	1445.4	887.482	1.17206

80	Furans	2,5-Dimethylfuran	C625865	C6H8O	96.1	894.2	243.994	1.03084
81	Benzenes	Toluene	C108883	C7H8	92.1	1057.2	334.151	1.02124

MW: molecular weight; RI: retention index; Rt: retention time; Dt: drift time; D: Dimer; M: Monomer

Table S2. Composition of volatile compounds in ten *A. arguta* fruits

Category	No.	Compounds	Aroma descriptors[a]	Volatile compounds Content of *A. arguta* fruit （μg/kg）									
				'Kuilv'	'Fenglv'	'Jialv'	'Wanlv'	'Xinlv'	'Pinglv'	'Lvbao'	'Cuiyu'	'Tianxinbao'	'Longcheng No2'
Aldehydes	1	(E)-2-Heptenal D	Almond, Fat, Fruit	8.25±0.79^{cde}	18.76±1.31^{a}	10.04±1.12^{bcd}	8.08±0.52^{cde}	10.59±0.3^{bc}	11.83±0.8^{b}	6.66±0.32^{de}	5±0.35^{e}	15.79±3.09^{a}	7.42±0.74^{cde}
	2	(E)-2-Heptenal M	Almond, Fat, Fruit	76.82±13.23^{c}	102.94±3.3^{ab}	92.07±1.55^{b}	96.06±0.68ab	97.95±2.56^{ab}	55.42± 3.3^{d}	37.03±1.53^{e}	39.71± 0.84^{e}	106.84±4.22^{a}	71.16±2.97^{c}
	3	(E)-2-Hexenal D	Apple	4201.45±200.38^{b}	5184.5±72.13^{a}	4378.17±153.11^{b}	3415.07±87.03^{c}	4499.6±44.58^{b}	3770.63±90.74^{c}	1224.19±42.5^{e}	1127.91±12.55^{e}	2584.37±140.94^{d}	2562.7±33.49^{d}
	4	(E)-2-Hexenal M	Apple	2736.77±122.44^{b}	2887.35±28.47^{ab}	2929.11±41.4^{a}	2548.6±30.06^{c}	2772.73±18.82^{ab}	2715.64±19.98^{bc}	1007.46±71.16^{f}	1108.74±11.34^{f}	1661.77±50.7^{e}	1931.08±27.97^{d}
	5	(E)-2-Octenal D	Dandelion, Fat, Fruit, Grass, Green, Spice	17.71±1.13^{d}	18.04±1.7^{d}	15.51±0.92^{d}	16.09±0.85^{d}	16.54±0.62^{d}	47.28±5.47^{b}	36.02±1.73^{c}	40.61±0.53^{bc}	43.39±4.92^{b}	61.75±2.76^{a}
	6	(E)-2-Octenal M	Dandelion, Fat, Fruit, Grass, Green, Spice	17.1±1.13^{d}	19.37±2.25^{d}	13.04±1.08^{d}	14.5±0.8^{d}	25.75±3.61^{d}	84.36±8.54^{b}	68.18±6.64^{c}	77.2±3.52^{bc}	83.99±8.71^{b}	111.99±4.37^{a}
	7	(E)-2-Pentenal D	Fruit, Pungent	166.59±7.57^{de}	431.85±12.57^{a}	268.46±18.87^{b}	199.21±6.01^{c}	277.62±22.83^{b}	149.15±9.62^{e}	38.58±4.13^{g}	52.06±3.36^{g}	192.95±15.23^{cd}	114.99±5.2^{f}
	8	(E)-2-Pentenal M	Fruit, Pungent	141.1±16.74^{cd}	177.12±1.99^{a}	175.32±2.04^{a}	133.36±0.47^{cd}	150.64±2.78^{bc}	162.66±5.66^{ab}	24.7±1.36^{f}	60.44±5.4^{e}	137.49±3.26^{cd}	132.29±7.28^{d}
	9	(Z)-4-Heptenal D	Dairy	21.85±2.85^{d}	20.94±1.88^{d}	23.42±0.48^{d}	34.29±1.26b^{cd}	29.73±2.29^{cd}	24.42±0.7^{d}	117.68±19.2^{a}	47.57±10.23^{bc}	55.08±7.55^{b}	51.65±8.7^{b}
	10	(Z)-4-Heptenal M	Dairy	12.96±1.53^{d}	17.79±1.43^{cd}	20.5±1.1b^{cd}	21.37±0.39^{bcd}	23.61±1.15^{bc}	26.97±1.14^{b}	80.32±8.86^{a}	15.64±3.28^{cd}	20.43±3.01^{bcd}	23.28±3.01^{bc}
	11	1-Hexanal D	Apple, Fat, Fresh, Green, Oil	1532.78±125.43^{b}	1911.11±45.8^{a}	1880.49±81.52^{a}	1849.91±32.17^{a}	1994.87±89.5^{a}	1178.76±54.06^{c}	193.47±24.52^{e}	250.34±22.55^{e}	1056.63±110.37^{c}	841.97±108.79^{d}
	12	1-Hexanal M	Apple, Fat, Fresh, Green, Oil	520.69±49.55^{ab}	520.59±14.12^{ab}	561.97±9.49^{a}	485.65±1.95^{b}	479.79±10.89^{b}	474.39±13.72^{b}	135.6±26.82^{e}	202.29±10.73^{d}	380.49±7.35^{c}	377.15±26.45^{c}
	13	1-Nonanal	Fat, Floral, Green, Lemon	32.21±2.38^{cd}	38.59±0.86^{ab}	30.55±0.71^{cd}	41.48±2.44^{a}	38.77±2.02^{ab}	34.19±0.41^{bc}	24.79±0.82^{ef}	19.88±1.17^{f}	28.14±1.12^{de}	21.63±1.56^{f}
	14	3-Methyl butanal	Fruit, Cheese	26.83±3.56^{g}	113.82±47^{c}	103.46±1.96^{cd}	155.76±6.9^{b}	192.14±1.29^{a}	169.08±9.39^{ab}	40.47±4.36^{ef}	56.56±7.41^{ef}	71.94±0.9^{de}	99.1±7.48^{cd}
	15	Valeraldehyde	loral, Pungent, Solvent	53.41±1.19^{cd}	62.95±2.57^{a}	61.01±0.62^{ab}	48.69±1.45^{d}	55.35±1.03^{bcd}	59.39±3.58^{abc}	38.81±2.46^{e}	36.95±2.78^{e}	37.86±2.99^{e}	40.59±4.44^{e}
	16	Benzaldehyde	Bitter Almond, Burnt Sugar, Cherry, Malt, Roasted Pepper	14.45±1.65^{ab}	10.99±0.25^{cde}	10.29±1.53^{def}	12.61±0.23^{bcd}	16.2±0.83^{a}	12.19±0.58^{bcd}	12.75±1.22^{bcd}	7.97±0.71^{f}	13.68±1.28^{abc}	8.35±0.53^{ef}
	17	Butanal D	Banana, Green, Pungent	2.93±0.55^{b}	4.43±0.56^{b}	5.36±0.28^{b}	8.83±0.69^{b}	8.45±0.6^{b}	7.87±1.22^{b}	35.14±0.65^{a}	15.74±0.67^{b}	9.41±0.91^{b}	34.86±6.28^{a}
	18	Butanal M	Banana, Green, Pungent	22.42±5.81^{a}	21.3±2.93^{ab}	19.15±1.57^{ab}	17.15±0.8^{ab}	18.3±1.73^{ab}	20.93±0.63^{ab}	15.92±3.58^{ab}	16.05±2.32^{ab}	10.36±0.63^{b}	15.71±3.9^{ab}

	19	Diethyl acetal	Creamy, Fruit, Pleasant, Tropical Fruit	142.5±17.62^{b}	124.64±13.76^{b}	138.61±10.25^{b}	111.27±15.95^{b}	139.77±16.71^{b}	129.62±11.02^{b}	188.32±14.93^{a}	214.09±10.02^{a}	121.8±4.28^{b}	140.67±4.96^{b}
	20	Heptanal D	Citrus, Fat, Green, Nut	21.81±0.46^{cd}	37.59±3.04^{bcd}	23.27±1^{cd}	39.5±2.24^{bc}	39.31±4.21^{bc}	20.89±2.55^{cd}	68.46±18.46^{a}	22.68±3.42^{cd}	49.92±7.02^{ab}	18.48±0.67^{d}
	21	Heptanal M	Citrus, Fat, Green, Nut	45.93±3.92^{de}	76.33±3.58^{a}	57.67±0.84^{cd}	59.38±1.78^{bc}	69.7±6^{ab}	53.17±5.06^{cd}	24.24±1.21^{f}	22.12±1.58^{f}	62.53±8.89^{bc}	39.67±3.37^{e}
	22	Valeraldehyde D	Almond, Bitter, Malt, Oil, Pungent	135.32±21.28^{bc}	88.07±14.04^{de}	154.99±21.56^{b}	159.78±8.71^{b}	101.35±6.09^{d}	56.91±5.43^{e}	202.09±8.84^{a}	66.8±4.64^{e}	115.91±6.06^{cd}	55.71±5.48^{e}
	23	Valeraldehyde M	Almond, Bitter, Malt, Oil, Pungent	183.68±5.21^{a}	149.16±10.4^{bc}	182.25±4.52^{a}	161.02±4.23^{b}	141.39±3.92^{c}	103.33±4.52^{d}	23.54±1.48^{f}	87.36±1.96^{e}	94.52±4^{de}	83.75±3.67^{e}
		Subtotal		10135.59	12038.21	11154.69	9637.65	11200.15	9369.09	3644.42	3593.72	6955.3	6845.95
		Percentage		62.93%	66.49%	69.41%	62.05%	64.90%	61.42%	16.77%	26.21%	37.96%	41.89%
	1	Isoamyl alcoholD	Burnt, Cocoa, Floral, Malt	184.35±5.31^{de}	192.75±6.11^{cd}	165.23±4.93^{def}	126.05±1.36^{f}	133.44±3.56^{f}	142.98±2.44^{ef}	281.58±9.91^{b}	228.15±3.33^{c}	343.2±7.73^{a}	207.26±6.94^{cd}
	2	(Z)-2-Penten-1-ol	ND	50.46±2.96^{c}	75.2±4.23^{ab}	55.39±2.2^{c}	60.7±1.25^{bc}	64.29±0.41^{bc}	72.78±2.81^{b}	62.35±0.85^{bc}	65.33±2.29^{bc}	90.28±13.65^{a}	89.94±1.88^{a}
	3	Isoamyl alcoholM	Burnt, Cocoa, Floral, Malt	114.43±8.61^{d}	79.91±5.48^{e}	77.08±2.47^{e}	64.17±2.47^{e}	58.8±0.14^{e}	65.56±1.15^{e}	188.86±3.79^{b}	236.88±3.29^{a}	189.46±8.83^{b}	148.68±4.29^{c}
	4	1-Hexanol D	Banana, Flower, Grass, Herb	75.45±3.78^{de}	66.06±6.85^{de}	49.09±3.46^{e}	48.54±0.64^{e}	96.82±2.75^{d}	91.5±6.9^{de}	447.47±26.2^{b}	358.49±17.27^{c}	431.6±9.11^{b}	498.76±22.36^{a}
	5	1-Hexanol M	Banana, Flower, Grass, Herb	333.43±7.55^{de}	308.05±9.6^{ef}	252±15.18^{g}	259.58±1.64^{fg}	379.85±12.94^{d}	382.39±15.93^{d}	911.98±21.3^{b}	810.94±37.17^{c}	902.87±3.51^{b}	997.55±21.79^{a}
	6	1-Hydroxy-2-propanone	Butter, Herb, Malt, Pungent	23.17±3.11^{c}	15.98±0.74^{d}	16±0.87^{d}	17.98±0.42^{cd}	13.42±0.93^{d}	18.16±0.35^{cd}	54.76±4.54^{a}	40.82±2.67^{b}	23.49±2.67^{c}	23.51±2.46^{c}
Alcohols	7	1-Octen-3-ol	Cucumber, Earth, Fat, Floral, Mushroom	49.28±3.21^{cd}	85.63±5.97^{b}	46.36±1.69^{cde}	77.47±4.25^{b}	103.94±3.66^{a}	77.58±4.56^{b}	42.04±3.87^{de}	35.93±1.91^{e}	54.76±1.96^{c}	46±3.71^{cde}
	8	1-Pentanol	Balsamic, Fruit, Green, Pungent, Yeast	116.83±6.49^{e}	111.16±4.12^{e}	128±6.58^{e}	211.74±4.04^{c}	162.76±1.58^{d}	132.04±3.58^{e}	277.36±7.24^{b}	395.48±11.46^{a}	291.69±6.67^{b}	288.82±7.18^{b}
	9	1-Penten-3-ol	Butter, Fish, Green, Oxidized, Wet Earth	61.23±4.55^{f}	176.68±3.5^{ab}	113.84±2.91^{e}	127.06±5.87^{de}	149.14±2.89^{c}	165.75±1.68^{b}	124.25±11.8^{de}	133.41±4.99^{cd}	188.44±10.37^{a}	127.07±4.62^{de}
	10	1-Propanol	Alcohol, Candy, Pungent	28.07±0.7^{c}	33.76±2.1^{c}	51.32±0.38^{b}	36.93±3.15^{c}	29.55±0.08^{c}	40.79±5.3^{bc}	28.23±1.74^{c}	89.73±0.86^{a}	86.53±3.2^{a}	77.28±6.5^{a}
	11	3-Methyl-2-butanol	Fruit	75.82±5.71^{bc}	115.71±6.59^{a}	57.81±0.87^{d}	81.31±1.52^{b}	118.5±3.81^{a}	52.3±4.24^{de}	44.26±2.99^{e}	22.87±3.55^{f}	40.15±3.51^{e}	64.43±4.81^{cd}
	12	1-Butanol	Fruit	54.51±3.68^{ef}	46.13±0.61^{fg}	50.58±0.42e^{fg}	62.61±0.6^{de}	43.86±1.65^{fg}	90.9±1.18^{b}	36.74±6.62^{g}	119.27±5.89^{a}	76.65±3.83^{cd}	79.03±7.48^{bc}
	13	Ethanol D	Alcohol, Brandy	503.51±15.31^{ab}	384.83±13.82^{cd}	306.86±15.9^{de}	307.21±14.35^{de}	290.7±32.93^{e}	358.88±11.34^{cde}	433.33±45.05^{bc}	540.43±17.44^{a}	346.1±63.36^{cde}	403.04±54.58^{c}

	14	Ethanol M	Alcohol, Brandy	169.63±2.01^{abc}	182.62±9.41^{a}	177.29±9.89^{ab}	180.02±3.52^{ab}	152.66±12.98^{abc}	174.48±3.76^{ab}	140.63±11.66^{c}	180.2±10^{ab}	150.06±7.63^{bc}	178.26±10.65^{ab}
	15	2-Heptanol	Citrus, Earth, Fried, Mushroom, Oil	64.35±7.5^{de}	49.23±3.33^{de}	34.75±2.74^{e}	32.74±2.29^{e}	49.98±8.06^{de}	36.03±2.72^{e}	137.26±12.58^{c}	96.51±14.47^{cd}	369.29±61.73^{a}	205.81±15.78^{b}
	16	Isobutanol D	Apple, Bitter, Cocoa, Wine	37.97±0.67^{cd}	25.63±2^{d}	27.61±1.34^{d}	33.54±2.89^{d}	22.72±1.28^{d}	33.46±0.57^{d}	182.64±14.96^{a}	51.25±2.55^{c}	49.78±3.34^{c}	70.78±9.21^{b}
	17	Isobutanol M	Apple, Bitter, Cocoa, Wine	28.43±5.61^{d}	12.44±0.67^{de}	14.47±0.83^{de}	11.7±0.31^{e}	12.77±1.34^{de}	20.57±1.49^{de}	147.57±12.53^{a}	87.74±6.39^{b}	28.28±5.76^{d}	56.19±10.44^{c}
		Subtotal		1947.74	1945.78	1607.69	1721.37	1869.8	1937.99	3486.54	3452.6	3639.13	3538.89
		Percentage		12.09%	10.75%	10.00%	11.08%	10.84%	12.71%	16.04%	25.18%	19.86%	21.66%
ketones	1	1-Penten-3-one D	Fish, Green, Mustard, Pungent	236.13±22.33^{d}	560.86±2.47^{a}	427.38±20.49^{b}	387±16.28^{b}	428.85±5.01^{b}	311.72±13.78^{c}	60.72±2.13^{e}	67.43±8.76^{e}	286.69±5.86^{c}	219.29±4.19^{d}
	2	1-Penten-3-one M	Fish, Green, Mustard, Pungent	147.95±9.1^{c}	199.12±3.29^{a}	202.43±4.48^{a}	168.97±3.18^{b}	175.51±2.35^{b}	169.51±6.29^{b}	38.22±3.96^{e}	57.21±6.48^{e}	132.48±0.86^{cd}	117.74±8.34^{d}
	3	Acetoin D	Butter, Creamy, Green Pepper	42±6.96^{de}	33.32±2.83^{e}	28.75±1.44^{e}	31.54±0.61^{e}	29.68±0.79e	30.12±2.79e	387.3±9.27^{b}	733.31±21.3^{a}	110.98±6.01^{d}	204.06±6.11^{c}
	4	Acetoin M	Butter, Creamy, Green Pepper	147.63±3.38^{d}	68.72±3.53^{e}	77.7±2.7^{e}	72.03±0.45^{e}	63.37±0.75^{e}	67.74±2.16^{e}	428.76±5.81^{b}	632.68±11.27^{a}	254.15±6.91^{c}	368.02±3.86^{b}
	5	3-Octanone	Butter, Herb, Mold	23.08±1.92^{de}	17.8±1.21^{e}	19.19±1.19^{e}	54.5±2.65^{a}	23.65±1.84^{de}	20.43±1.41^{de}	39.36±7.5^{bc}	29.87±3.07^{cd}	49.34±6.73^{ab}	50.1±2.67^{a}
	6	Acetone	Pungent	374.59±12.93^{c}	464.58±6.51^{a}	408.25±9.96^{b}	368.18±5.92^{cd}	412.38±15.88^{b}	340.82±3.29^{d}	197.73±14.25^{ef}	229.32±8.35^{e}	181.46±12.01^{f}	212.68±23.45^{ef}
	7	Pentan-2-one	Fruit, Pungent	286.9±23.01^{e}	518.62±66.54^{bc}	236.12±30.79^{e}	398.32±7.02^{d}	516.63±45^{bc}	766.05±29.47^{a}	432.29±51.52^{cd}	709.28±22.4^{a}	716.1±49.86^{a}	569.82±31.5^{b}
		Subtotal		1281.44	1879.02	1415.82	1498.51	1663.49	1724.54	1639.12	2499.92	1754.7	1765.21
		Percentage		7.96%	10.38%	8.81%	9.65%	9.64%	11.31%	7.54%	18.23%	9.58%	10.80%
Acids	1	Acetic acid D	Acid, Fruit, Pungent, Sour, Vinegar	25.89±0.79^{bc}	30.74±2.18^{a}	23.23±0.71^{cd}	29.81±0.96^{a}	31.35±0.46^{a}	28.62±0.71^{ab}	21.09±1.82^{d}	23.76±1.1^{cd}	25.45±1.06^{bc}	24.04±1.29^{cd}
	2	Acetic acid M	Acid, Fruit, Pungent, Sour, Vinegar	581.64±8.58^{c}	206.58±8.75^{d}	167.85±4.93^{d}	300.49±2.05^{d}	246.41±5.53^{d}	216.06±6.93^{d}	1503.48±73.47^{a}	790.14±21.43^{b}	1322.26±100.7^{a}	811.06±102.91^{b}
		Subtotal		607.53	237.32	191.08	330.3	277.76	244.68	1524.57	813.89	1347.71	835.1
		Percentage		3.25%	1.16%	1.06%	1.81%	1.41%	1.39%	6.05%	4.68%	6.21%	4.25%
Esters	1	Ethyl acetate	Aromatic, Brandy, Grape	254.1±2.06^{abc}	236±0.48^{d}	238.3±3.85^{d}	248.68±4.69^{bcd}	243.33±3.87^{cd}	240.63±3.29^{cd}	262.13±11.76^{ab}	267.26±4.34^{a}	235.47±2.73^{d}	235.4±4.95^{d}
	2	Propyl acetate	Celery, Floral, Pear, Red Fruit	5.53±0.63^{b}	6.68±0.77^{b}	6.8±0.47^{b}	8.24±0.69^{b}	7.85±0.39^{b}	8.19±0.62^{b}	222.56±9.24^{a}	14.56±0.57^{b}	32.53±8.08^{b}	15.24±0.56^{b}
	3	Methyl benzoate	Herb, Lettuce, Prune, Violet	6.82±0.13^{c}	8.86±0.45^{c}	6.91±0.37^{c}	8.77±0.84^{c}	12.42±1.16^{a}	9.21±1.24^{bc}	6.98±0.15^{c}	6.94±0.25^{c}	6.98±1.28^{c}	11.53±1.19^{ab}
	4	Ethyl butyrate D	Apple, Butter, Cheese, Pineapple, Strawberry	74.25±1.89^{b}	51.32±9.35^{c}	22.58±1.72^{de}	55.27±1.76^{c}	105.41±10.45^{a}	30.87±5.37^{d}	103.83±13.03^{a}	16.32±2.78^{de}	12.08±0.65^{e}	53.44±5.94
	5	Ethyl butyrate M	Apple, Butter, Cheese, Pineapple, Strawberry	73.15±8.03^{ab}	76.17±7.47^{ab}	44.57±1.63^{bcd}	78.37±0.86^{ab}	99.54±3.21^{a}	55.66±4.19^{bcd}	27.68±3.37^{d}	31.21±5.88^{cd}	64.57±6.36^{abc}	59.76±8.26^{bcd}

6	Butyl isovalerate D	Fruity	126.38±7.72^{c}	129.57±3.89^{c}	118.96±0.41^{c}	131.65±7.17^{c}	114.63±5.33^{c}	108.01±4.17^{c}	2179.86±130.74^{a}	466.64±87.95^{c}	1158.67±111.06^{b}	365.65±37.51^{c}
7	Butyl isovalerate M	Fruity	87.38±4.57^{d}	98.44±3.75^{cd}	67.57±3.11^{d}	80.02±4.4^{d}	86.82±8.29^{d}	68.37±6.81^{d}	512.06±44.49^{a}	187.77±9.19^{bc}	493.79±46.23^{a}	250.74±48^{b}
8	Butyl acetate D	Apple, Banana	55.85±2.7^{e}	258.3±40.44^{bc}	148.52±1.78^{cde}	59.04±1.78^{e}	202.97±15.59^{bcd}	273.57±36.74^{b}	550.77±122.76^{a}	162.26±27.97^{bcde}	131.86±13.06^{de}	82.66±8.26^{de}
9	Butyl acetate M	Apple, Banana	105.3±1.45^{f}	172.51±6.05^{de}	168.06±1.52^{de}	107.44±2.27^{f}	173.53±2.03^{d}	195.9±4.26^{c}	422.17±3.81^{a}	237.94±8.83^{b}	154.99±1.51^{e}	158.75±4.49^{de}
10	Ethyl formate D	Pungent	4.86±1.63^{c}	12.45±1.1abc	12.79±0.35^{abc}	17.67±0.58^{ab}	20.53±0.85^{ab}	17.2±2.23^{ab}	16.8±1.16^{ab}	10.33±4.06^{bc}	22.27±1.38^{a}	18.66±8.3^{ab}
11	Ethyl formate M	Pungent	58.49±0.85^{b}	55.85±4.33^{b}	57.77±0.55^{b}	71.28±2.72^{a}	53.42±3.51^{b}	69.55±4.1^{a}	40.12±1.4^{c}	38.45±1.94^{c}	53.16±1.79^{b}	51.54±4.31^{b}
12	Geranyl formate	Floral	25±1.72^{cde}	28.09±0.43^{bc}	20.24±0.8^{ef}	32.07±2.91^{ab}	36.47±3.02^{a}	26.92±2.68^{bcd}	20.95±2.97^{def}	17.9±1.97^{f}	33.18±2.52^{ab}	19.83±1.86^{ef}
13	Hexyl acetate	Apple, Banana, Grass, Herb, Pear	28.16±5.25^{cde}	19.85±1.25^{e}	21.08±0.84^{e}	21.33±2.01^{e}	20.06±0.72^{de}	22.03±1.51^{e}	81.01±6.91^{a}	30.66±3.68^{bcd}	38.48±1.94^{b}	31.14±2.29^{bc}
14	Hexyl propanoate	Apple, Banana, Grass, Herb, Pear	62.36±14.35^{b}	25.92±2.23^{cd}	19.66±1.44^{cd}	18.9±0.47^{d}	17.8±0.5^{d}	16.37±1.27^{d}	27.78±3.82^{cd}	45.94±2.04^{bc}	54.61±8.95^{b}	117.14±13.96^{a}
15	Isopentyl acetate D	Apple, Banana, Glue, Pear	75.79±9.52^{b}	56.92±0.67^{b}	57.64±1.23^{b}	59.95±3.22^{b}	67.33±2.57^{b}	56.46±2.06^{b}	2501.48±138.88^{a}	255.38±97.67^{b}	260.85±56.92^{b}	106.78±24.54^{b}
16	Isopentyl acetate M	Apple, Banana, Glue, Pear	66.84±1.77^{e}	71.76±0.7^{e}	64.26±2.1^{e}	67±3.16^{e}	66.92±1.86^{e}	63.82±3.31^{e}	506.74±25.93^{a}	303.25±57.17^{b}	223.16±12.51^{c}	148.92±15.48^{d}
17	Isobutyl acetate	Apple, Banana, Floral, Herb	35.36±5.47^{b}	40.73±1.54^{b}	42.12±2.63^{b}	41.02±2.73^{b}	40.94±0.5^{b}	30.49±2.13^{b}	2074.07±151.52^{a}	164.56±5.61^{b}	32.46±1.82^{b}	175.04±14.69^{b}
18	Methyl isobutyrate	Flower, Fruit	135.22±17.73^{bc}	179.78±10.09^{a}	122.84±5.23^{c}	130.35±5.09^{bc}	151.77±8.43^{b}	128.76±5.87^{bc}	84.96±3.52^{d}	65.69±3.58^{d}	189.39±3.03^{a}	130.64±12.26^{bc}
19	Methyl acetate	Ester, Green	82.35±9.64^{d}	53.39±3.19^{d}	49.93±1.03^{d}	57.41±1.48^{d}	65.46±3.71^{d}	49.64±1.6^{d}	408.04±49.53^{b}	237.51±32.16^{c}	565.05±60.86^{a}	332.55±60.64^{b}
20	Methyl butanoate	Apple, Banana, Cheese, Ester, Floral	28.21±3.2^{e}	37.24±2.94^{cd}	32.58±1^{de}	63.66±0.9^{a}	43.73±1.16^{bc}	62.94±0.6^{a}	11.29±1.81^{f}	49.1±4.46^{b}	68.03±3.3^{a}	62.56±3.11^{a}
21	Pentyl acetate D	Apple, Banana, Glue, Pear	28.65±0.74^{c}	30.41±3.43^{c}	29.97±2.04^{c}	40.41±8.35^{c}	32.26±3.45^{c}	31.93±3.45^{c}	550.87±39.83^{a}	150.2±14.43^{bc}	258.69±15.65^{b}	78.4±4.81^{c}
22	Pentyl acetate M	Apple, Banana, Glue, Pear	29.84±1.07^{d}	31.12±0.8^{d}	28.25±0.34^{d}	36.87±2.18^{d}	30.96±2.77^{d}	27.59±0.18^{d}	333.02±11.98^{a}	212.79±16.67^{b}	235.57±17.64^{b}	115.8±14.97^{c}
23	Ethyl propionate	Apple, Pineapple, Rum, Strawberry	2.26±0.12^{b}	2.46±0.2^{b}	2.97±0.06^{b}	2.67±0.06^{b}	2.38±0.08^{b}	2.67±0.41^{b}	5.5±1.18^{b}	3.25±0.61^{b}	16.86±3.36^{a}	4.14±0.78^{b}
24	Propyl propionate	Apple, Banana, Pineapple	12.49±3.16^{b}	12.55±1.16^{b}	11.48±1.2^{b}	12.7±1.33^{b}	19.95±2.73^{b}	7.85±0.84^{b}	194.33±17.11^{a}	10.8±3.75^{b}	6.12±0.36^{b}	15.19±2.21^{b}
	Subtotal		1464.64	1696.37	1395.84	1450.78	1716.49	1604.62	11145	2986.71	4348.81	2641.47
	Percentage		9.09%	9.37%	8.69%	9.34%	9.95%	10.52%	51.28%	21.78%	23.74%	16.16%

Terpenes	1	Terpinolene	Pine	251.65±24.54^{a}	35.43±5^{de}	18.73±1.16^{e}	205.38±17.94^{b}	106.19±18.4^{c}	29.9±4.45^{e}	48±9.39^{de}	76.86±9.01^{cd}	28.25±2.46^{e}	200.48±30.56^{b}
	2	α-Phellandrene	Citrus, Fresh, Mint, Pepper, Spice, Wood	59.95±2.94^{de}	26.97±0.78^{e}	32.85±1.29^{de}	261.67±13.79^{a}	140.65±7.47^{c}	32.92±1.18^{de}	70.3±11.63^{d}	29.73±3.06^{e}	46.23±3.29^{de}	196.78±4.68^{b}
	3	α-Pinene	Cedarwood, Pine, Sharp	21.01±6.65^{ab}	4.44±0.38^{c}	4.55±0.19^{c}	25.2±4.03^{a}	12.18±2.65^{bc}	8.37±1.62^{c}	4.79±0.72^{c}	11.22±0.91^{bc}	9.3±0.66^{c}	20.93±7.42^{ab}
	4	Myrcene	Balsamic, Fruit, Geranium, Herb, Must	31.67±2.21^{c}	19.66±0.69^{c}	18.76±1.12^{c}	136.27±14.1^{a}	19.37±2.31^{c}	67.8±4.76^{b}	23.58±0.7^{c}	26.99±2.24^{c}	23.44±4.13^{c}	30.59±6.51^{c}
	5	β-Pinene	Pine, Polish, Wood	111.86±13.576^{a}	25.4±0.98^{d}	21.75±1.77^{d}	83.24±10.67^{b}	44.61±5.71^{c}	31.14±3.35^{cd}	28.5±1.71^{cd}	30.79±2.21^{cd}	24.46±1.4^{d}	95.24±3.59^{ab}
		Subtotal		476.13	111.89	96.64	711.76	323.01	170.13	175.17	175.6	131.68	544.02
		Percentage		2.96%	0.62%	0.60%	4.58%	1.87%	1.12%	0.81%	1.28%	0.72%	3.33%
Pyrazines	1	2,3,5-Trimethylpyrazine	Cocoa, Earth, Must, Potato, Roast	43.37±1.73^{a}	16.23±0.97^{bc}	13.44±0.46^{bcd}	14.01±0.59^{bcd}	15.11±1.59^{bcd}	10.28±0.27^{bcd}	7.15±0.53^{cd}	6.39±0.39^{d}	9.58±0.9^{bcd}	18.61±2.48^{b}
		Subtotal		43.37	16.23	13.44	14.01	15.11	10.28	7.15	6.39	9.58	18.61
		Percentage		0.27%	0.09%	0.08%	0.09%	0.09%	0.07%	0.03%	0.05%	0.05%	0.11%
Furans	1	2,5-Dimethylfuran	Savory	108.12±8.18^{bc}	137.33±7.22^{a}	139.87±8.02^{a}	113.21±5.6^{b}	144.21±10.78^{a}	137.19±3.61^{a}	105.06±5.24^{bc}	134.29±5.61^{a}	93.85±5.92^{c}	108.13±1.14^{bc}
		Subtotal		108.12	137.33	139.87	113.21	144.21	137.19	105.06	134.29	93.85	108.13
		Percentage		0.67%	0.76%	0.87%	0.73%	0.84%	0.90%	0.48%	0.98%	0.51%	0.66%
Benzenes	1	Toluene	ND	41.73±4.18^{c}	44.4±1.68^{bc}	54.99±1.6^{a}	54.05±5.22^{a}	46.35±1.39^{bc}	54.96±0.94^{a}	6.55±0.36^{d}	50.24±2.55^{ab}	41.29±1.25^{c}	43.39±1.44^{c}
		Subtotal		41.73	44.4	54.99	54.05	46.35	54.96	6.55	50.24	41.29	43.39
		Percentage		0.26%	0.25%	0.34%	0.35%	0.27%	0.36%	0.03%	0.37%	0.23%	0.27%
		Total		16106.28	18106.55	16070.06	15531.64	17256.37	15253.48	21733.59	13713.35	18322.04	16340.77

Means with different letters in the same column indicate significant differences (Duncan's test, $p < 0.05$). Note: a From Flavornet database (https://www.femaflavor.org; https://foodb.ca/compounds; http://www.flavornet.org; accessed August 15, 2023). ND: Not reported in relevant literature.

Table S3. Sample information of *A. arguta.*

No.	Variety	Selection time and place	Selection methods	Harvest time
1	‘Kuilv’	Selected from wild resources in 1980 from Fuxing Forestry, Ji'an County, Jilin Province.	asexual reproduction	September 6, 2022
2	‘Fenglv’	Selected from the wild resources of Fuxing Forest Farm, Ji'an County, Jilin Province, 1980.	asexual reproduction	September 6, 2022
3	‘Jialv’	Selected from wild resources in Huanren, Liaoning, 1984.	asexual reproduction	September 6, 2022
4	‘Wanlv’	Selected from the wild resources of Zuojia Township, Jilin City, 1997.	asexual reproduction	September 13, 2022
5	‘Xinlv’	Selected in 1968 from wild resources in Zuojia Township, Jilin City.	asexual reproduction	September 6, 2022
6	‘Pinglv’	Selected from wild resources of Yulin Commune, Ji'an County, Jilin Province, 1981.	asexual reproduction	September 6, 2022
7	‘Lvbao’	A good single wild plant was found in 1981 in the cold onion ditch of Dunhua, Jilin.	asexual reproduction	September 6, 2022
8	‘Cuiyu’	Wild resources collected in 1980 from Waifanggou, Liangshui Commune, Ji'an County, Jilin Province.	asexual reproduction	September 6, 2022
9	‘Tianxinbao’	Selected from 24 mixed live progeny of germplasm resources in Zuojia Township, Jilin City, China.	vegetative reproduction	September 6, 2022
10	‘Longcheng No2’	Selected from wild resources in Kandian County, Denning, 2002.	asexual reproduction	September 13, 2022

Table S4. Overview of the sampling sits.

The sampling sit	Longitude	Latitude	Average temperature (°C)	Average precipitation (mm)	Average air pressure (hPa)	Active cumulative temperature (°C)	Average relative humidity (%)	Average wind speed (m/s)
Actinidia arguta Resource Nursery of the Institute of Special Animal and Plant Sciences of the Chinese Academy of Agricultural Sciences in Zuojia Town	44°00′-44°07′	126°01′-126°-08′	5.6	679	991.6	2779.8	67	2.6

Table S5. Organic acid standard curves.

Name	Standard Curves	R^2
Oxalic Acid	Y=15184x+28.758	0.9994
Malic Acid	Y=917.66x+19.027	0.9995
Shikimic Acid	Y=65935x+474.72	0.9995
Lactic Acid	Y=683.52x-60.731	0.9997
Citric Acid	Y=1487x-4.2267	0.9998
Quinic Acid	Y=611.85x-0.598	0.9998

Table S6. Analysis conditions.

Gas-ion transport spectral unit	Automatic headspace injection unit

Analysis time	30 min	Inlet volume	100 μL
Column type	MXT-WAX（30 m×0.53× 1 μm）	Incubation time	10 min
Column temperature	60℃	Incubation temperature	60℃
Carrier Gas / Drift Gas	N_2 (99.999% pure)	Injection needle temperature	85℃
IMS temperature	45℃	Incubation speed	500 rpm

Table S7. Gas chromatography conditions.

Time	E1（Drift Gas）	E2（Carrier Gas）	Recording
00:00,000		2 mL/min	rec
02:00,000		2 mL/min	-
10:00,000	150 mL/min	10 mL/min	-
20:00,000		100 mL/min	-
30:00,000		100 mL/min	Stop
